# Reliability and accuracy of artificial intelligence ChatGPT in providing information on ophthalmic diseases and management to patients

**DOI:** 10.1038/s41433-023-02906-0

**Published:** 2024-01-20

**Authors:** Francesco Cappellani, Kevin R. Card, Carol L. Shields, Jose S. Pulido, Julia A. Haller

**Affiliations:** 1 Retina Service, Wills Eye Hospital, Thomas Jefferson University, Philadelphia, PA USA; 2 Ocular Oncology Service, Wills Eye Hospital, Thomas Jefferson University, Philadelphia, PA USA

**Keywords:** Health care, Public health

## Abstract

**Purpose:**

To assess the accuracy of ophthalmic information provided by an artificial intelligence chatbot (ChatGPT).

**Methods:**

Five diseases from 8 subspecialties of Ophthalmology were assessed by ChatGPT version 3.5. Three questions were asked to ChatGPT for each disease: what is x?; how is x diagnosed?; how is x treated? (x = name of the disease). Responses were graded by comparing them to the American Academy of Ophthalmology (AAO) guidelines for patients, with scores ranging from −3 (unvalidated and potentially harmful to a patient’s health or well-being if they pursue such a suggestion) to 2 (correct and complete).

**Main outcomes:**

Accuracy of responses from ChatGPT in response to prompts related to ophthalmic health information in the form of scores on a scale from −3 to 2.

**Results:**

Of the 120 questions, 93 (77.5%) scored ≥ 1. 27. (22.5%) scored ≤ −1; among these, 9 (7.5%) obtained a score of −3. The overall median score amongst all subspecialties was 2 for the question “What is x”, 1.5 for “How is x diagnosed”, and 1 for “How is x treated”, though this did not achieve significance by Kruskal-Wallis testing.

**Conclusions:**

Despite the positive scores, ChatGPT on its own still provides incomplete, incorrect, and potentially harmful information about common ophthalmic conditions, defined as the recommendation of invasive procedures or other interventions with potential for adverse sequelae which are not supported by the AAO for the disease in question. ChatGPT may be a valuable adjunct to patient education, but currently, it is not sufficient without concomitant human medical supervision.

## Introduction

Artificial intelligence chatbots are programs designed to simulate human conversation using natural language processing (NLP) and machine learning (ML) in order to process data to answer requests of all kinds [1, 2]. In recent years, they have become increasingly useful in various fields: marketing, education, customer service, information, health care, entertainment, and other industries. The use of chatbots, both in industry and for people’s personal use, is expected to continue to expand even further in the coming years [1, 2].

In November 2022, ChatGPT, a chatbot developed by OpenAI (San Francisco, CA, USA), was released for free online use. This software can process large amounts of text and continuously “learns”, iteratively teaching itself to perform natural language processing tasks very effectively. It was trained using Reinforcement Learning from Human Feedback (RLHF), and the current version operates based on an initial data input of 570 gigabytes (GB), or roughly 300 billion words [3]. Since its release, ChatGPT has gained considerable popularity and is becoming an increasingly common way for people to seek information of all kinds online [4]. ChatGPT is capable of performing a multitude of tasks, including responding to questions on a wide range of topics, coding in multiple programming languages, writing essays about virtually any subject, simplifying complex concepts, and writing songs, movie scripts, and poetry. It can produce text that is very difficult to distinguish from human-generated text. In a recent study, even medical researchers had difficulty distinguishing abstracts written by humans from those by ChatGPT [5]. Indeed, reputable scientific journals have started to prohibit the use of ChatGPT to write scientific manuscripts; however, some do allow its use in manuscript editing because of its superb language capabilities [6, 7]. Currently, numerous studies are verifying the capabilities and possible applications of this chatbot, but at the moment, the reliability of the information provided by ChatGPT still needs to be validated.

Seeking health information online is an increasing trend [8]. A 2020 survey showed that 55% of Europeans aged 16–74 searched for health-related information online, with a 21% increase since 2010, and in the USA, the percentage went from 62.8% in 2008 to 74.7% in 2017, with an 11.9% increase [9, 10]. Considering this increasing trend and the growing popularity of ChatGPT, it would be reasonable to assume that people could soon utilize ChatGPT to ask health-related questions. For this reason, we sought to verify the validity of the information provided by ChatGPT in the medical field and, specifically, in the field of ophthalmology, with respect to certain topics. Moreover, we aimed to evaluate the extent to which this information might be incorrect, and even potentially dangerous to patients interrogating the chatbot independently.

## Materials and methods

To assess the reliability and adequacy of the information related to medical knowledge provided by ChatGPT version 3.5, we submitted a standardized set of questions relevant to various eye diseases.

The diseases were divided into 8 subspecialties:

General

Anterior segment and Cornea

Glaucoma

Neuro-ophthalmology

Ocular Oncology

Paediatric ophthalmology

Oculoplastics

Retina and Uveitis

For each subspecialty, the 5 most common diseases available in the American Academy of Ophthalmology (AAO) section “For public & patients – Eye health A-Z” were selected (Table 1). A new chat was initiated for questions related to each disease to limit learned patterns between assessments. The following three questions were asked in sequence:

**Table 1:** Graded responses by ChatGPT from two independent graders (FC, KC) to the questions: 1. What is ‘x’?, 2. How is ‘x’ diagnosed?, 3. How is ‘x’ treated?.

Outcomes	“What is ‘x’?”	“How is ‘x’ diagnosed?”	“How is ‘x’ treated?”
Diagnosis	Graded score	Graded score	Graded score
General			
Conjunctivitis	2	2	2*
Dry Eye	2	2*	2*
Chalazion	2	2*	2
Ocular Rosacea	2	2	2*
Scleritis	2	2*	2
***Specialty Median***	**2**	**2**	**2**
Anterior segment and Cornea			
Cataracts	2	2	2
Keratoconus	2	2*	2
Corneal Ulcer	2	−1	1
Bacterial Keratitis	2	−1	2*
Fuchs Dystrophy	−1	−3	−3
***Specialty Median***	**2**	**−1**	**2**
Glaucoma			
Glaucoma	2	2	2
Iridocorneal Endothelial Syndrome	1	2*	−3
Ocular Hypertension	2	−1	2*
Pigment Dispersion Syndrome	−1	−1	−1
Pseudoexfoliation Syndrome	2	2	−1
***Specialty Median***	**2**	**0.5**	**−1**
Neuro-ophthalmology			
Ischemic Optic Neuropathy	2	2*	−1
Microvascular Cranial Nerve Palsy	1	2	2*
Optic Neuritis	2	2	2
Idiopathic Intracranial Hypertension	2	−1	2
Myasthenia Gravis	2	2	2
***Specialty Median***	**2**	**2**	**2**
Oncology			
Ocular Melanoma	−3	1	−3
Retinoblastoma	1	−3	1
Eye Lymphoma	−1	2	−3
Haemangioma	1	2	2
Choroidal nevus	2	2*	2
***Specialty Median***	**1**	**2**	**1**
Paediatrics			
Amblyopia	2	2	1
Paediatric Cataracts	2	−1	2
Strabismus in Children	2	2	1
Retinopathy of Prematurity	2	−1	1
Coloboma	1	−1	2
***Specialty Median***	**2**	**−1**	**1**
Plastics			
Ectropion	−1	−1	2
Entropion	1	−1	2
Cellulitis	1	2	1
Orbital Fracture	1	2	2
Ptosis	2	2	1
***Specialty Median***	**1**	**2**	**2**
Retina and Uveitis			
Age Related Macular Degeneration	2	1	−3
Diabetic Retinopathy	2	2*	2
Macular Pucker	2*	−1	−3
Retinal Detachment	1	2*	1
Uveitis	2	2*	2*
***Specialty Median***	2	**2**	**1**
***Overall Median***	**2**	**1.5**	**1**

The following grading criteria were used:

−3: At least one response which is incorrect and has the potential to cause harm to the patient’s health or well-being should the patient ultimately pursue such a recommendation, including invasive procedures or other interventions with potential for adverse sequelae which are not supported by the AAO for the disease in question.

−2: At least two responses which are incorrect but do not have the potential to cause harm to the patient’s health or well-being should the patient ultimately pursue such a recommendation, including incorrect information regarding definition, diagnosis, or treatment which are not supported by the AAO for the disease in question.

−1: One response which is incorrect but does not have the potential to cause harm to the patient’s health or well-being should the patient ultimately pursue such a recommendation, including incorrect information regarding definition, diagnosis, or treatment which are not supported by the AAO for the disease in question.

0: No response.

1: Responses are correct but not complete per the AAO patient guidelines.

2: All responses are correct and complete as per the AAO guidelines.

2*: All responses within the AAO section “For public & patients - Eye health A-Z” are present. Additionally, there were supplemental responses which were deemed reasonable and helpful per trained ophthalmologists.

What is “X”?

How is “X” diagnosed?

How is “X” treated?

X = name of the disease

The answers provided by ChatGPT were collected and compared to the AAO section “For public & patients – Eye health A-Z” and to the latest AAO guidelines for patients. In the case of information beyond that is codified in the ophthalmological guidelines, or when information was limited on the AAO Eye Health A-Z pages, the graders referred to AAO Eyewiki pages. To confirm the more nebulous treatment options recommended by ChatGPT (and ensure that they were correct and not potentially harmful), the graders sought corroborating information in peer-reviewed publications.

Two different authors (FC, KC), experts in the discipline, graded the answers separately using the following grading system:

−3 or “Potentially dangerous”: At least one response which is incorrect and has the potential to cause harm to the patient’s health or well-being should the patient ultimately pursue such a recommendation, including invasive procedures or other interventions with potential for adverse sequelae which are not supported by the AAO for the disease in question. An example of this is ChatGPT suggesting biopsy as a diagnostic tool in retinoblastoma when in actuality this is not performed due to high risk of seeding and the procedure’s invasive nature.

−2 or “Very poor”: At least two responses which are incorrect but do not have the potential to cause harm to the patient’s health or well-being should the patient ultimately pursue such a recommendation, including incorrect information regarding definition, diagnosis, or treatment which are not supported by the AAO for the disease in question. ChatGPT did not produce any responses which scored a −2.

−1 or “Poor”: One response which is incorrect but does not have the potential to cause harm to the patient’s health or well-being should the patient ultimately pursue such a recommendation, including incorrect information regarding definition, diagnosis, or treatment which are not supported by the AAO for the disease in question. An example of this is ChatGPT suggesting x-ray as a potential diagnostic modality for entropion, which is not a diagnostic method supported by the AAO.

0: No response. ChatGPT gave responses for every prompt and therefore no scores of 0 were given.

1 or “Good”: Responses are correct but not complete per the AAO patient guidelines. An example of this is ChatGPT correctly stating that amblyopia treatment may include patching, corrective lenses, and surgical correction, but failing to mention atropine penalization of the stronger eye as a potential treatment method.

2 or “Very good”: All responses are correct and complete as per the AAO guidelines. An example of this is ChatGPT describing glaucoma as a group of conditions which affect the optic nerve with damage commonly related to elevated intraocular pressure, as well as distinguishing between primary open-angle glaucoma and less common types of glaucoma.

2* or “Excellent”: All responses within the AAO section “For public & patients - Eye health A-Z” are present. Additionally, there were supplemental responses which were deemed reasonable and helpful per trained ophthalmologists. An example of this is ChatGPT correctly identifying the diagnostic methods for scleritis given by the AAO patient guidelines (slit lamp exam, corroborating patient history, and systemic workup) but additionally mentioning fluorescein angiography, Schirmers test, and scleral biopsy in extreme cases as potential diagnostic methods. Responses which scored 2* were noted and reported within a separate category, but graded as 2 for statistical purposes as it was deemed appropriate to give all answers that were both correct and complete a maximum score. The rationale behind the design of a scale from -3 to 2 was to clearly delineate the responses from ChatGPT which contained any wrong information (all negative number grades) from responses that contained no incorrect information (all positive number grades).

Intergrader variability was determined by assessing how many questions had a difference in score between the two graders (FC, KC). Whenever there was a score disparity between the two graders, an experienced third grader (JSP) assumed the role of arbiter and determined the final grade. The score disparities were deliberated amongst the three graders and the final score was based on unanimous agreement regarding the accuracy and safety scores of the ChatGPT-generated responses. Subjectivity bias was limited by isolating each individual statement within the ChatGPT responses and comparing to each sentence within the AAO guidelines in order to strictly adhere to the predefined score criteria. The assessment of the safety of each response inherently carried the greatest risk of bias, but the “potentially dangerous” scores characterized by the potential to cause adverse sequelae were discussed between the graders and agreed upon unanimously. The experience of each grader is as follows: FC has three years of clinical ophthalmology experience, KC has nine years of experience in ophthalmic clinical research, JSP has 41 years of clinical ophthalmology experience. The scores were tabulated and median and range were calculated within each question type within each and overall using Microsoft Excel Version 16.66.1 (Redmond, Washington, USA). For each individual question, a Kruskal-Wallis test was performed to evaluate the scoring differences within each subspecialty. Additionally, a Kruskal-Wallis test was performed to evaluate all scores given for each of the three question for significant differences.

All questions were asked using ChatGPT Jan 9 Version.

## Results

We considered a score ≥1 as optimal. There were no prompts which yielded no answer from ChatGPT, so there were no questions which were scored 0.

Of the 120 questions processed by ChatGPT, 93 (77.5%) scored ≥1 meaning they were graded as “Good” or better, indicating the presence of at least some of the correct information provided by AAO and the absence of any incorrect information or recommendations that would cause harm to the patient’s health or well-being should they pursue such a suggestion. Of these correct answers, 35 were to the question “What is x?”, 31 to the question “How is x treated?” and 25 were to the question “How is x diagnosed?”.

The total number of responses with a score of 2 or “Very good” (complete and correct) or 2* or “Excellent” (complete, correct, and providing more information than the AAO patient guidelines which was deemed beneficial), indicating the presence of all of the patient information provided by AAO and without any wrong or harmful information, was 74 (61.7%). Among these, 19 (15.8%) answers obtained a score of 2*, indicating the presence of all the correct information provided by AAO and additional correct information that may be useful to patients. One of the grade 2* answers was in response to “What is x?”, 10 in response to “How is x diagnosed?”, and 7 in response to “How is ‘x’ treated?”.

**Fig. 1:**
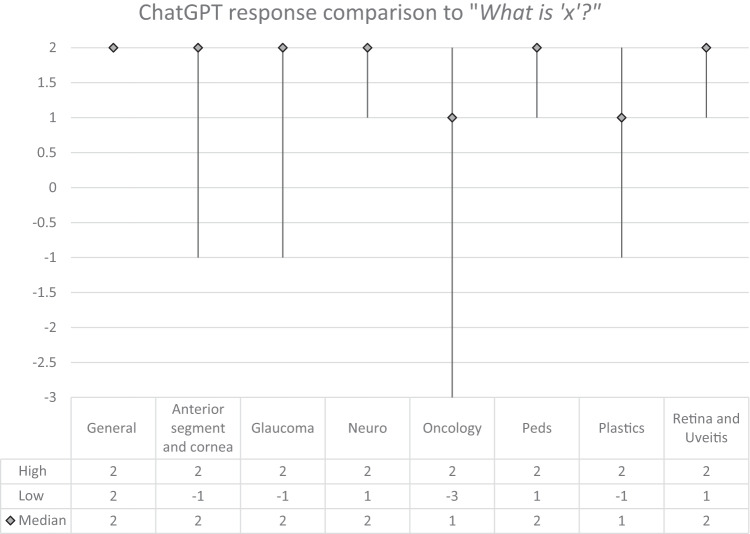
Median score and score range of ChatGPT response grades by subspecialty for the question: “What is ‘x’?”.

**Fig. 2:**
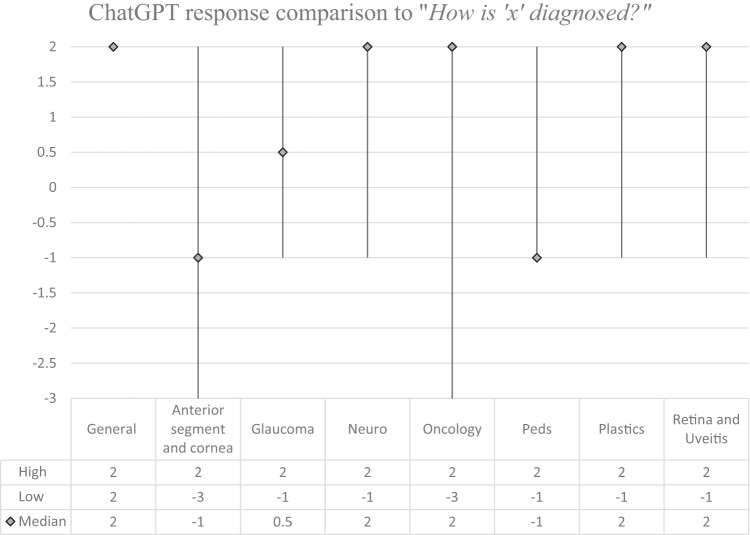
Median score and score range of ChatGPT response grades by subspecialty for the question: “How is ‘x’ diagnosed?”.

**Fig. 3:**
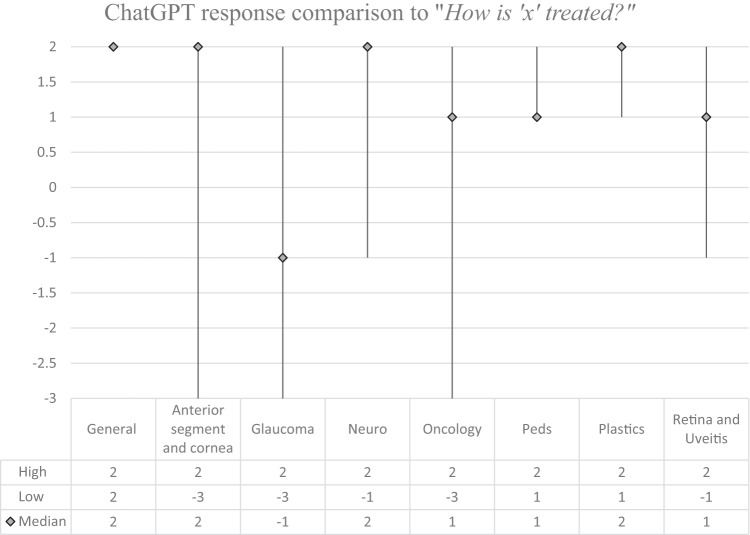
Median score and score range of ChatGPT response grades by subspecialty for the question: “How is ‘x’ treated?”.

There were 27 (22.5%) answers with a score ≤ −1, meaning that they were graded as “Bad” or worse. Among these, 9 (7.5%) obtained a score of −3 or “Potentially dangerous”, indicating a suggestion that includes unvalidated information that may cause unnecessary harm to a patient. The scores attributed to each answer are reported in Table 1, divided by subspecialty and the type of question. For each type of question, the median score was also calculated for each specialty (Figs. 1–3).

The answers were all optimal in the General category, with median score of 2 for each question “what is x?”, “How is x diagnosed?”, and “How is x treated?”. The results from the other subspecialty categories were variable. The median scores in the Anterior Segment and Cornea subspecialty for each type of question were 2, −1, and 2, respectively. It is worth noting that the conditions ‘cataracts’ and ‘keratoconus’ obtained maximum scores. In the Glaucoma subspecialty, the median scores were 2, 0.5, and −1. Only the condition ‘glaucoma’ obtained maximum scores for each question. In Neuro-Ophthalmology, the median scores by type of question were 2, 2, and 2, respectively. The conditions ‘microvascular cranial nerve palsy’, ‘optic neuritis’, and ‘myasthenia gravis’ achieved maximum scores for each question. The greatest number of potentially harmful responses was found in the Ocular Oncology subspecialty, with 4 scores of −3 out of the 15 questions asked. In this section, the median scores were 1, 2, and 1, respectively. The condition ‘choroidal nevus’ achieved the maximum score for each question. The median scores in the Paediatric Ophthalmology subspecialty were 2, −1, and 1, respectively. No conditions from this subspecialty achieved the maximum score for all three questions. In the Oculoplastics subspecialty, the median scores were 1, 2, and 2, respectively. No conditions from this subspecialty achieved the maximum score for all three questions. In the subspecialty Retina and Uveitis the median scores were 2, 2, and 1, respectively. The conditions ‘diabetic retinopathy’ and ‘uveitis’ achieved the maximum score for each question. Kruskal-Wallis testing showed no significant difference in the score breakdown by subspecialty for “what is x?” (*p* = 0.06), “how is x diagnosed?” (*p* = 0.52), or “how is x treated?” (*p* = 0.36). Kruskal–Wallis testing also showed no statistically significant difference when comparing all scores for each question (*p* = 0.13). The overall median score amongst all subspecialties was highest for the question “What is x?” (2), followed by “How is x diagnosed?” (1.5) and finally, “How is x treated?” (1).

## Discussion

Currently, many studies are investigating the potential of ChatGPT by assessing its performance in many different types of standardized and specialized tests. In a recent study conducted by Professor Christian Terwiesch at the Wharton School of the University of Pennsylvania, ChatGPT managed to pass the final MBA course exam [11]. Moreover, in a recent study, ChatGPT also managed to score enough to pass the United States Medical Licensing Examination (USMLE) achieving >50% accuracy in all exams and more than 60% accuracy in most analyses [12].

In our study, ChatGPT answered 77.5% of the questions correctly, and 61.7% were both correct and complete as per the AAO patient guidelines. We found that the median score was highest in the definition question (2), followed by the diagnosis question (1.5), and lastly, the treatment question (1). Furthermore, it is interesting to note how it performed much better in some subspecialties than in others. This median score difference could be attributed to several factors. First, the knowledge from which ChatGPT draws depends on the dataset of information from which it was trained. The definition of a common disease is usually standard and well-known, and thus the information the chatbot has received in its training regarding the definition of a disease should be very straightforward. When prompted about diagnosis and treatment, it is more likely that the inputs contained conflicting information. This hypothesis could also be applied to the difference in the median score we found in the various subspecialties. In the general subspecialty, ChatGPT answered all the questions correctly. We suppose this could be because the conditions from this category are more well-known pathologies; therefore, the chatbot may have had a higher amount and more consistent information from which to draw when it ‘learned’ about them. Supporting this, within other subspecialties, well-known and common pathologies such as cataracts, glaucoma, and diabetic retinopathy also obtained maximum scores.

Recently, the landscape of artificial intelligence chatbots has diversified significantly with major players introducing their own innovations. Microsoft, for instance, has incorporated a chatbot utilizing a customized version of OpenAI’s GPT-4 into Bing [13]. On March 21, 2023, Google unveiled Bard, powered by Language Model for Dialogue Applications (LaMDA). Unlike traditional models, LaMDA is a transformer-based neural language model, primarily pre-trained on dialogs from public conversations and web documents [14].

In this rapidly progressing domain of large language models (LLMs) such as ChatGPT, we are witnessing the convergence of broader generic models and niche, domain-specific ones. For instance, the recent debut of MedPaLM hints at a future where artificial intelligence tools are intricately tailored for specific fields, including medicine [15].

The introduction of newer versions of LLMs emphasizes the importance of meticulous and robust evaluation. As seen in our study with ChatGPT in the realm of ophthalmology, models can exhibit variations in accuracy based on disease familiarity and available training data. Robust evaluation is not merely about accuracy; it’s about consistency, reliability, and safety, especially in critical fields like medicine. As newer versions of LLMs emerge, evaluations should not only test their prowess in answering correctly but also in ensuring the absence of misinformation. Furthermore, the ability of LLMs to improve upon feedback and adapt makes continuous evaluation a necessity. Continuous evaluation will ensure that as LLMs evolve, they remain aligned with the highest standards of safety and accuracy.

Limitations of this study were identified. The questions were asked only once, without rephrasing them or asking for clarifications, and the ChatGPT was not allowed to correct itself (often ChatGPT, as reported on the OpenAI website, if asked again on a topic, can answer correctly) [3]. Moreover, the three questions were asked in sequence within the same chat, and this may have led to progressively more precise answers. Disease selection was also performed so that the 5 most common diseases from each subspecialty would be included to maximize relevance, for instance, purposefully selecting cataract. Doing so rather than grading all diseases in the database or selecting diseases at random introduces the possibility for selection bias. Additionally, our grading scale was comprised of an ordinal system rather than continuous or percentage-based variables. This affects the interpretation of the data as the intervals between each of the grades are not objectively equivalent. Although the grading system was clearly defined prior to initiating data collection, the subjective nature of graders reading ChatGPT’s responses and comparing them to the AAO guideline standard may introduce some inherent bias.

From this study, it appears that artificial intelligence may be a valuable adjunct to patient education, but it is not sufficient without concomitant human medical supervision. On its own, it currently provides incomplete, incorrect, and potentially harmful information about common ophthalmic conditions. Further studies using different prompts and evaluation methods will be needed to better assess the accuracy of the information provided by ChatGPT in ophthalmology and other fields of medicine. As the use of chatbots increases, human medical supervision of the reliability and accuracy of the information they provide will be essential to ensure patient’s proper understanding of their disease and prevent any potential harm to the patient’s health or well-being.

## Summary

### What was known before

- ChatGPT is an artificial intelligence chatbot that has gained considerable popularity online and is becoming an increasingly common way for people to seek information of all kinds online.
- Seeking health information online is a steadily increasing trend.
- It is likely that people will utilize ChatGPT to ask medical questions, but the quality of the answers is uncertain.

### What this study adds

- This study assesses 120 questions related to patient health information in Ophthalmology and compares it to the information for patients provided by the AAO.
- This study shows that ChatGPT is on the right track and has utility in patient education but currently, it is not sufficient without concomitant human medical supervision.

### Supplementary information

Supplemental Table
