# Supplementary material for: Reliability and accuracy of artificial intelligence ChatGPT in providing information on ophthalmic diseases and management to patients
Source: Eye (Lond). 2024 Jan 20;38(7):1368–73. doi: 10.1038/s41433-023-02906-0 (PMC11076805; doi:10.1038/s41433-023-02906-0)
Supplement: Supplementary file 1 — Supplemental Table [file 41433_2023_2906_MOESM1_ESM.docx]

Supplemental figure: Examples of chatGPT responses that received different scores. Note that the score of -2 was not assigned to any response.

| **Question** | **Answer** | **Score and comment** |
| --- | --- | --- |
| **How is retinoblastoma diagnosed?** | Retinoblastoma is typically diagnosed through a combination of techniques, including:  A physical examination of the eye, including a dilated eye exam to check for signs of the tumor.  Imaging tests such as an ultrasound, CT scan, or MRI, which can help to confirm the presence of the tumor and determine its size and location.  A biopsy, in which a small sample of tissue is taken from the eye and examined under a microscope to confirm the diagnosis.  Fluorescein angiography, in which a special dye is injected into the bloodstream and the eye is examined with a special camera to detect any abnormal blood vessels that may be associated with the tumor.  Electroretinography (ERG) which is a test that measure the electrical activity in the retina, it is used to detect retinoblastoma.  It is important to note that early diagnosis is crucial for successful treatment of retinoblastoma. Therefore, any parents or caregivers who suspect their child may have retinoblastoma should seek medical attention as soon as possible. | -3  The recommendation for a biopsy in the context of retinoblastoma is inaccurate and poses a significant risk. Biopsies are infrequently indicated for retinoblastoma due to the high potential of tumor seeding. This erroneous suggestion could lead to harm if followed. |
| **What is pigment dispersion syndrome?** | Pigment Dispersion Syndrome (PDS) is a condition in which small pigment particles, called lens epithelium-derived cells (LECs), are released from the iris (the colored part of the eye) into the fluid inside the eye. This can cause the iris to become more pigmented and can lead to the formation of a cloudy area in the front of the eye, called a glaucoma. PDS is associated with increased intraocular pressure, which can cause damage to the optic nerve and lead to vision loss if left untreated. It is a relatively rare condition. | -1  While there is accurate information presented on PDS, chatGPT incorrectly states that the “formation of a cloudy area in the front of the eye” is “called a glaucoma.” |
| **How is retinopathy of prematurity treated?** | Retinopathy of prematurity (ROP) is typically treated with laser therapy or cryotherapy. The goal of treatment is to prevent the abnormal blood vessels from causing further damage to the retina. In some cases, the treatment may also involve a surgery called vitrectomy, which is used to remove scar tissue from the retina. The treatment plan will vary depending on the severity of the condition and the stage of ROP. Close monitoring is also important to ensure that the condition does not worsen. | 1  While the provided information regarding retinopathy of prematurity was correct, there was an omission of anti-VEGF therapy in the discussion which is mentioned in the AAO guidelines for patients. As a result, we considered the answer correct but not complete. |
| **What is keratoconus?** | Keratoconus is a degenerative disorder of the eye in which the cornea, the clear front surface of the eye, thins and bulges outward into a cone-like shape. This can cause distorted vision, glare, and sensitivity to light. It is typically diagnosed in adolescence and progresses until the late twenties or early thirties, and then stabilizes. Keratoconus can be treated with glasses, contact lenses, or surgery depending on the severity of the condition. | 2  The definition provided for Keratoconus was accurate, concise, and complete when compared to the AAO patient guidelines. |
| **How is scleritis diagnosed?** | Scleritis is typically diagnosed through a combination of a thorough eye examination, patient history, and additional testing. The eye examination will include an evaluation of the sclera for signs of inflammation, such as redness, swelling, and tenderness. The ophthalmologist may also perform tests such as a Schirmer's test, which measures the amount of tears produced by the eye, or a fluorescein angiogram, which uses a dye and special camera to take pictures of the blood vessels in the eye. Additionally, the doctor may also order blood tests to look for underlying autoimmune disorder, or other systemic conditions associated with Scleritis. In some cases, a biopsy of the sclera may be performed to confirm the diagnosis and rule out other causes of eye inflammation. The diagnosis of Scleritis is important to be done as soon as possible, as it can be a serious condition that can cause vision loss or eye damage if left untreated. | 2*  ChatGPT accurately listed diagnostic methods for scleritis. Additionally, fluorescein angiography, Schirmer test, and scleral biopsy in extreme cases were presented as supplemental diagnostic options, which were not mentioned in the AAO patient guidelines. |
